# Polylactic Acid Chemical Foaming Assisted by Solid-State Processing: Solid-State Shear Pulverization and Cryogenic Milling

**DOI:** 10.3390/polym14214480

**Published:** 2022-10-22

**Authors:** Philip R. Onffroy, Nathan T. Herrold, Harrison G. Goehrig, Kalie Yuen, Katsuyuki Wakabayashi

**Affiliations:** Department of Chemical Engineering, Bucknell University, Lewisburg, PA 17837-2029, USA

**Keywords:** solid-state shear pulverization, cryogenic milling, polylactic acid, foams, processing, semicrystalline polymers, compression molding

## Abstract

A chemical foaming process of polylactic acid (PLA) was developed via the solid-state processing methods of solid-state shear pulverization (SSSP) and cryogenic milling. Based on the ability of solid-state processing to enhance the crystallization kinetics of PLA, chemical foaming agents (CFA) are first compounded before foaming via compression molding. Specifically, the effects of the pre-foaming solid-state processing method and CFA concentration were investigated. Density reduction, mechanical properties, thermal behavior, and cell density of PLA foams are characterized. Solid-state processing of PLA before foaming greatly increases the extent of PLA foaming by achieving void fractions approximately twice that of the control foams. PLA’s improved ability to crystallize is displayed through both dynamic mechanical analysis and differential scanning calorimetry. The solid-state-processed foams display superior mechanical robustness and undergo low stress relaxation. The cell density of the PLA foams also increases with solid-state processing, especially through SSSP. Additionally, crosslinking of PLA during the pre-foaming processing step is found to result in the greatest enhancement of crystallization but decreased void fraction and foam effectiveness. Overall, SSSP and cryogenic milling show significant promise in improving chemical foaming in alternative biopolymers.

## 1. Introduction

Polymer foams have widespread valuable applications, including packaging, safety padding, and insulation [1]. Polymer foams are created by incorporating pressurized gas into a molten polymer and subsequently solidifying the polymer-gas composite. In the case of semicrystalline polymers, gas is captured both by entanglement in the polymer chains and by polymer crystallites [2,3,4]. Today, nearly all polymers in commercial foams are derived from non-renewable fossil fuels and do not degrade easily [5]. Their ubiquitous use can be an environmental challenge. In the pursuit of developing bio-based and/or biodegradable polymers to replace petroleum-based polymers in foams, a variety of strategies have been taken, ranging from plant-based materials to microorganism-produced polymers [6,7,8,9]. One of the most studied bio-based polymers is polylactic acid (PLA), a condensation polymer derived through the fermentation of sucrose from cornstarch into lactic acid [10,11,12]. PLA, known to be more compostable than petroleum-based plastics in accordance with ASTM D6691 [13,14], is becoming a prevalent sustainable material of choice in biomedical, packaging, and additive manufacturing applications [11].

Polymer foams can be created through physical pressurized gas injection or by incorporating gas generated from chemical reactions. To date, most PLA-foaming studies with high levels of success are limited to the former physical foaming method [2,15,16]. However, physical foaming tends to produce unevenly distributed foams with less versatility in product shape than chemical foaming and can be expensive due to the need for high-pressure gas sources and precision transport systems [17,18]. Chemical foaming, which uses small molecular additives known as chemical foaming agents (CFAs) that break down into gas when heated above their activation temperature [2,19], can circumvent the physical foaming concerns and enable a thoroughly consistent, in situ foam through common polymer processing methods, such as extrusion, injection molding, and compression molding [3]. However, the limited success of chemical foaming of PLA has been reported to date [18,20,21].

Creating an effective PLA foam is challenging partly due to PLA’s slow crystallization kinetics, which allows the foaming gas to predominantly escape from the system rather than being secured by PLA’s crystalizing chains as the temperature cools to shape a product [2,22]. Additionally, PLA is shear-sensitive in the melt state, suffering from molecular, viscosity, and physical property degradation compared to petroleum-based analogs [2]. Incorporating other polymers, such as polyethylene into a blend with PLA overcomes these challenges, but significantly lessens the sustainable nature of the output [23]. Another potential solution is crosslinking PLA chains via chemical crosslinking agents; however, the crosslinking often still must be accompanied by blending PLA with another polymer such as poly(butylene succinate) to achieve an adequate foam [24].

In previous studies, an alternative processing method called solid-state shear pulverization (SSSP) has shown promising results in increasing the crystallization kinetics of PLA [25,26,27], which is considered key for better control of the foam cell structure [2]. SSSP is a form of twin screw extrusion conducted under chilled conditions, and it has previously been used to modify homopolymers [28,29], compatibilize polymer blends [30,31,32], disperse additives [33,34], and create nanocomposites [35,36,37,38,39]. The foci of SSSP have been at the forefront of polymer sustainability, ranging from mechanical recycling [40,41] and natural fiber/renewable feedstock composites [42,43,44], to PLA/starch blends [45] and PLA crystallinity studies [25,26]. Specifically, the mechanochemistry of SSSP leads to scission and imperfections in PLA chains, which increase the material’s rate of nucleation and growth [25]. Another solid-state processing method called cryogenic milling (cryomilling), has also been employed alongside SSSP [46,47,48] and has contributed to previous sustainable PLA processing research [49,50].

This is the first study in the literature to use SSSP and cryomilling to facilitate the chemical foaming of PLA, aiming to develop a more sustainable biopolymer foam. PLA foams are prepared by first incorporating a CFA with neat polymer pellets via a solid-state process, and subsequently compression-molding it into a specimen. These SSSP and cryomill techniques are compared to a control prepared via manual blending. An additional set of crosslinked PLA foams processed through cryomilling is introduced to investigate the combination of crosslinking and solid-state processing. Void fractions for the different sets of PLA foams are first measured. The foam morphology characterization through scanning electron microscopy (SEM) imaging is followed by thermal analysis of the foams via differential scanning calorimetry (DSC) and mechanical property evaluation with static compression testing and dynamic mechanical analysis (DMA). The processing-structure-property relationships of pre-foaming solid-state compounding of the CFA and the biopolymer are explored.

## 2. Materials and Methods

### 2.1. Materials

The PLA material used in this study was Ingeo Biopolymer 2003D with an L-lactide to D-lactide ratio of 96/4, supplied by NatureWorks, LLC [51]. This extrusion grade material is reported by the manufacturer to have a density of 1.24 g/cm^3^, a melt flow rate of 6 g/10 min at 210 °C, tensile yield strength of 60 MPa, and a heat distortion temperature of 55 °C [51]. Due to PLA’s hygroscopic nature, it was dried for at least 2 h at 90 °C in a Moretto XD1 Dryer before all procedures.

The CFA used for this study was an azodicarbonamide (ADCA)-based CFA custom formulated by Avient Corporation. This CFA came in viscous liquid form and its formulation consisted of 54 wt% ADCA with the remainder being carrier, active, surfactant, and clay thickener. This ADCA CFA activates and releases nitrogen (N_2_), carbon monoxide (CO), and ammonia (NH_3_) in an exothermic event [18]. The activation temperature of approximately 205 °C, determined via in-house thermogravimetric analysis testing, is key, as the activation temperature is higher than PLA’s measured melting point of around 170 °C; the higher activation temperature ensures that the PLA is molten and able to contain and dissolve the gas [52]. The crosslinking agents used in the final portion of this study were triallyl isocyanurate (TAIC) and dicumyl peroxide (DCP) [53], purchased from Sigma Aldrich.

### 2.2. Pre-Foaming Processing Methods

Both SSSP and cryomilling were used as the primary processing methods for compounding CFA with PLA before foaming. PLA pellets manually blended with CFA were designated as the third control formulation, modeling a traditional process where polymer pellets and additives were directly fed into a molding machine without any solid-state preprocessing step. The fourth formulation of crosslinked PLA was prepared by first crosslinking PLA through single-screw melt extrusion followed by cryomill-compounding with CFA. For the balance of this paper, the SSSP-processed foam set will be referred to as SP, the cryomill-processed set as CM, the melt blended control set as CT, and the crosslinked/cryomill-processed set as XL. For each of the four sets of pre-foam processing modes, a CFA content parametric study was carried out to determine the relationships between the weight percentage of CFA and the physical properties of the resulting foams. The nominal concentrations of CFA for the six series tested were 0.5 wt%, 1.0 wt%, 2.0 wt%, 3.5 wt%, 5.0 wt%, and 6.5 wt%.

**Figure 1:**
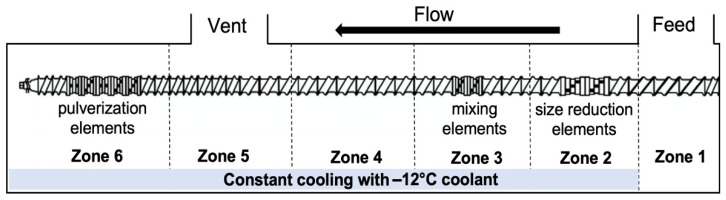
The SSSP screw design used in this study contains 9 bilobe kneading discs distributed among conveying elements for size reduction, mixing, and pulverization purposes.

For foam set SP, CFA was compounded with PLA pellets through SSSP. The SSSP processing method is based on a KraussMaffei Berstorff ZE25-UTX intermeshing, co-rotating twin screw extruder with a screw diameter of 25 mm and the length-to-diameter ratio of 34. The extruder barrels were chilled to low temperatures using a circulation of −12 °C-ethylene glycol/water solution, provided by Budzar Industries BWA-AC10 chiller. Figure 1 outlines the screw configuration, taken from a previous study [25], which employed a balance of harsh and mild screw elements to disperse the CFA additives while preventing premature polymer decomposition during the SSSP process. PLA pellets were manually coated in the liquid CFA and fed into the SSSP barrel using a Brabender Technologie Volumetric RotoTube feeder with the assistance of pressurized air through the center of the feeder hopper to ensure a continuous flow of 50 g/h. The SSSP screw speed was set to 200 rpm based on a previous parametric study on SSSP processing-structure-property relationships [54].

For foam set CM, the cryomill processing method achieved a similar low-temperature mechanochemical compounding effect as SSSP, in a batch setting [55]. Each cryomill run was composed of a 12-g total sample of PLA with CFA, run through a SPEX SamplePrep 6870 Freezer/Mill. The cryomill procedure started with a 15-min cooldown period followed by 5 cycles of 4 min of pulverization and 4 min of cooldown between each cycle. After the final cycle, the sample contents were thawed to room temperature and stored.

For control foam set CT, PLA pellets were manually blended with CFA with a 20-g total sample size in a glass container. This mixture was prepared and stored at room temperature.

Foam set XL followed a two-step preparation process. The first part involved melt-compounding PLA pellets with 0.1 wt% TAIC and 0.1 wt% DCP crosslinking agents through a Killion Model KLB075 single-screw extruder. The screw speed was set to 15 RPM, and an extruder temperature of 180 °C was used because that is above both the melting temperature of PLA and the activation temperature of the crosslinking agents. The crosslinked polymer extrudate was cooled to ambient temperature and pelletized. The second step was to compound the crosslinked PLA with CFA in a cryomill in the same manner as foam set CM.

### 2.3. Compression Molding Foaming Process

After the four pre-foaming preparation methods were completed, the foaming procedure was carried out in a consistent fashion using compression molding. A 5.0 g sample of each formulation was added into a custom, cylindrical stainless steel mold with a 7.6 cm inner diameter and 6.4 cm height. The mold was loaded into an automated Carver AutoFour 30-15 HC Press. Under an initial 5 MPa of pressure, the sample was pressed at 220 °C and held isothermally for 8 min; during this process, pressure increase was observed inside the mold as CFA activated between 190–210 °C. The pressure was released, and the mold was cooled at an average rate of approximately 10 °C/min on a steel cooling surface with convective air cooling from two AC Infinity Model AI-MPF120P2 dual fans. After at least 20 min of cooling and resting, the foam sample was removed from the mold and stored.

### 2.4. Foam Analysis Methods

The density reduction measurements of the foam samples were conducted following the ASTM D792 standard using an OHAUS Density Determination Kit and Adventurer Model AX324 scale. The density of the sample was first calculated as:

(1) $$\rho_{foam}=\frac{A}{A-B}\left(\rho_{0}-\rho_{L}\right)+\rho_{L}$$

where *A* is the weight of the sample in air, *B* is the weight of the sample in water, air density ($\rho_{L}$) equals 0.00119 g/cm^3^, and water density ($\rho_{0}$) equals 0.997 g/cm^3^ at 25 °C. The void fraction (ϕ) of the foam samples, which is the volume expansion ratio of the material caused by foaming [21,56], was then calculated using the following equation:

(2) $$\phi =\frac{V_{void}}{V_{sample}}=1-\frac{\rho_{foam}}{\rho_{PLA}}$$

In Equation (2), $\rho_{foam}$ is the density of the foam sample calculated via Equation (1), PLA density ($\rho_{PLA}$) equals 1.24 g/cm^3^, $V_{void}$ represents the volume taken up by gas cells inside the sample, and $V_{sample}$ is the overall volume of the sample.

Scanning electron microscopy (SEM) was conducted using a Hitachi SU 5000 Field Emission Scanning Electron Microscope. Surfaces of cryogenically fractured PLA foam samples were sputter-coated with gold using a Denton Desk IV. SEM images were taken under a high vacuum with an electron beam voltage of 3.0 kV and at a magnification of ×70. These SEM images were used to quantitatively compare the gas cell distribution in samples using the software program ImageJ [57]. The cell density (*N_C_*), defined as the number of cells per volume of non-foamed base PLA material, was calculated for each SEM micrograph following Equation (3):

(3) $$N_{C}={\left(\frac{n*M^{2}}{A}\right)}^{\frac{3}{2}}*\frac{1}{1-\phi }$$

In Equation (3), ϕ is the void fraction, *n* is the number of cells counted in each micrograph image, *A* is the cross-sectional area of the foam in the image, and *M* is the magnification factor of the image.

Differential scanning calorimetry (DSC) was performed on foam samples using a TA Instruments Q2000, calibrated with indium. A standard heat-cool-reheat run between 0 °C and 220 °C was programmed with a ramp rate of 10 °C/min.

Dynamic mechanical analysis (DMA) was conducted in a compression mode where a 12.7 mm diameter cylindrical cut-out of each foam sample was placed on a custom stainless-steel platform and subjected to oscillatory compressive stress by a cylindrical steel plunger. The compression deformation mode was chosen because it most closely resembles the mechanical strain a polymer foam material would undergo in applications such as packaging. Each compression DMA run was conducted at an oscillation frequency of 1 Hz in a dynamic temperature ramp mode between −20 °C and 170 °C. Additionally, static compression and stress relaxation runs for each sample were conducted at room temperature. The compression strain rate was 0.003/s and the initial static load for stress relaxation was set at 200 kPa [58].

## 3. Results and Discussion

### 3.1. Density Reduction

**Figure 2:**
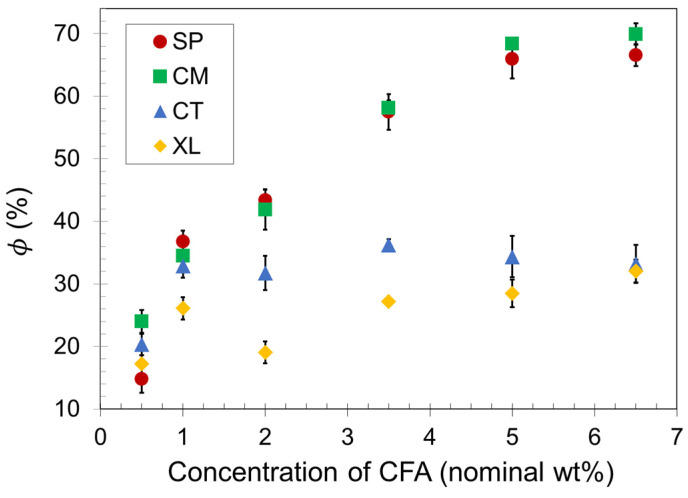
Void fraction of SSSP-processed (SP), cryomilled (CM), manually blended control (CT), and crosslinked/cryomilled (XL) PLA foam samples.

Density reduction measurements were conducted on the compression-molded samples to obtain average ϕ values, which are presented as a function of CFA concentration in Figure 2. As the CFA content increased, the void fraction increased for all samples up to a maximum plateau value. The plateau in each set indicates that there is an upper limit to the number of gas cells that a compression-molded PLA foam can successfully contain upon foam expansion, even as an increasing amount of gas is released inside the polymer melt. The different plateau values shown in Figure 2 for each of the four sets reveal that the solid-state processing of PLA before foaming makes a significant difference to the maximum void fraction a foam sample can achieve with compression molding. The manually blended control set CT reached a void fraction plateau of about 35% at a relatively low CFA concentration of 1.0 wt%, whereas the SSSP and cryomill sets (SP and CM, respectively) reached void fraction plateaus of approximately 70% at a CFA concentration of 5.0 wt%.

Polymer foaming technology often employs crosslinking to effectively capture gas cells and impart prototypical slow recovery foam behavior [59,60]. This study included a crosslinked analog of set CM to investigate the combination of crosslinking and cryogenic milling. Figure 2 reveals that the crosslinked foam set XL resulted in significantly lower void fraction values than set CM. PLA is a relatively brittle polymer at room temperature, and crosslinking may have constrained the chains of the material to such a great extent that fewer gas cells could be contained, as the XL set reached a void fraction plateau of approximately 30%.

### 3.2. Foam Morphology

**Figure 3:**
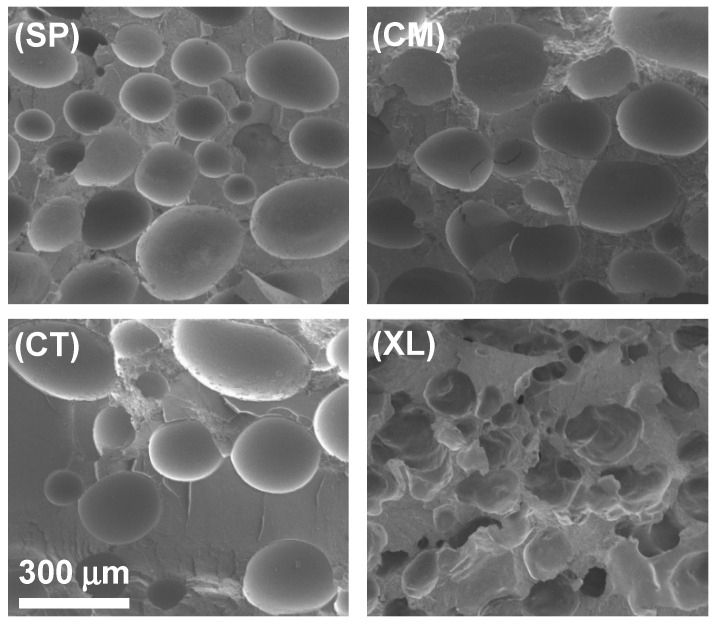
SEM images of SSSP-processed (SP), cryomilled (CM), manually blended control (CT), and crosslinked/cryomilled (XL) PLA foams with 6.5 wt% CFA.

The cross-sectional gas cell morphology was evaluated with SEM, for different concentrations of CFA across four pre-foaming processing methods. Figure 3 displays how the four processing methods resulted in different gas cell shapes and size distributions, in a representative comparison of the 6.5 wt% CFA loading series. The non-crosslinked, solid-state-processed foams in Figure 3(SP) and Figure 3(CM) showed similarly high areas of coverage by closed cells. The SP samples displayed smaller gas cells than the CM samples across different CFA concentrations despite similar cell area coverage and ϕ values from an earlier analysis. When comparing the SP and CM samples to the CT sample in Figure 3, it appears the control foam also exhibited closed cells. However, cell area coverage in CT foams was lower than those of the solid-state-processed foams, revealing one major reason why the control foams had lower void fraction values. In addition, the cells in the CT foams were concentrated in clusters around the sample rather than distributed evenly, for example, clustering at the top of Figure 3(CT).

A combination of the results so far indicates that PLA compounded with CFA in SP and CM methods were able to be compression-molded into consistent and physically expanded foams, containing a greater amount of gas in closed cells, compared to the CT foams. One explanation is that the mechanochemical modification of the PLA chains enabled enhanced crystallization kinetics [25], leading to a higher effect in trapping gas in closed cells upon solidification. Another explanation is that the intimate and homogeneous mixing in SSSP and cryomilling increased the CFA distribution and its contact level with PLA prior to the foaming process [36,37,42,47].

The crosslinked foams, such as shown in Figure 3(XL), displayed cross-sectional morphology significantly different from the other sets in that open cells were formed instead of closed cells. Open cell structure is a common characteristic of polymer foams with high void fractions [61]. Despite the apparent network structure and moderate open cell concentration, the XL foams did not expand in the mold as much as the SP and CM foams. This indicates that the crosslinking agents made the material too strong and tough to be able to contain as much gas as the other foams, contributing to its significantly lower ϕ values [62].

**Figure 4:**
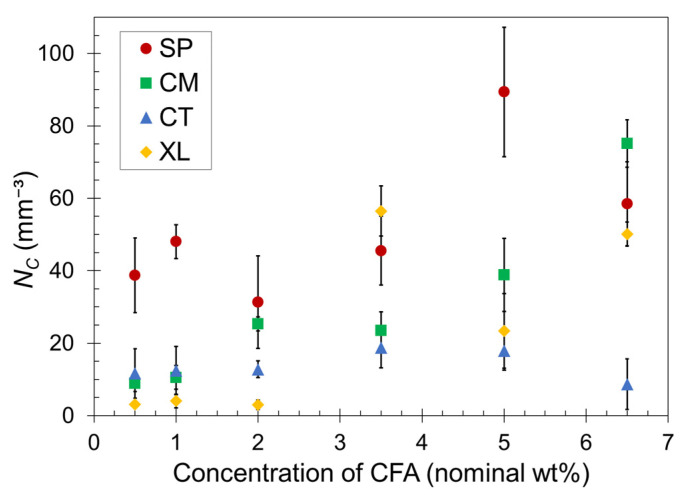
Calculated cell density versus CFA concentration for SP, CM, CT, and XL foams. The error bars indicate one standard deviation. The data points that do not show error bars have deviation ranges smaller than the point size.

Quantitatively, the cell density for each foam was calculated using Equation (3). The average *N_C_* values are plotted as a function of CFA concentration in Figure 4. For a given CFA concentration, the cell density values generally reflect the visual trends observed in the SEM images. However, the standard deviation ranges overlap for many data points in Figure 4, and therefore we refrain from making definitive remarks but rather provide general observed trends.

Both sets of solid-state-processed foams displayed greater cell density than the CT and XL foam sets in most cases, confirming the enhanced ability of the pre-foaming solid-state processing to generate and capture the gas in closed cells. The SP foams tended to have greater gas cell density than CM foams, particularly at low CFA concentrations. At higher CFA content, the CM set began to achieve similarly high *N_C_* values as the SP set. Perhaps the shearing nature of SSSP is more conducive to dispersing CFA than impact-based cryomilling at low concentrations. As CFA concentration increases, this nuanced difference becomes less relevant because the amount of gas being released is high and the effect of enhanced solidification rate dominates the level of CFA dispersion in these materials. The XL samples experienced the most inconsistent trend, with the majority of *N_C_* values remaining low except for high jumps observed for 3.5–6.5 wt% CFA. The inconsistency of the XL foam results can be attributed to the open-cell nature of the crosslinked foams causing less consistent cell formation compared to the other closed cell foams.

Lastly, it might be expected that the plateauing trend of ϕ in Figure 2 would correspond to a similarly plateauing trend of cell density in Figure 4. This may be occurring for the SP and CT sets but is not the case for the CM set. Perhaps the fine powder nature of CM formulations after cryomilling enabled gas cell formation more consistent with CFA content than other sets. In contrast, the XL samples showed a delayed increase in cell density while ϕ is relatively steady in Figure 3. Further investigation on the relationship between ϕ and cell density is warranted, but one definitive takeaway is that consistent PLA foams with practical density reduction are reliably achievable with CFA concentrations at around 5–6.5 wt%. These CFA loadings are considerably higher than a typical industry polymer foam CFA concentration of around 1.0 wt% [63].

### 3.3. Differential Scanning Calorimetry

**Figure 5:**
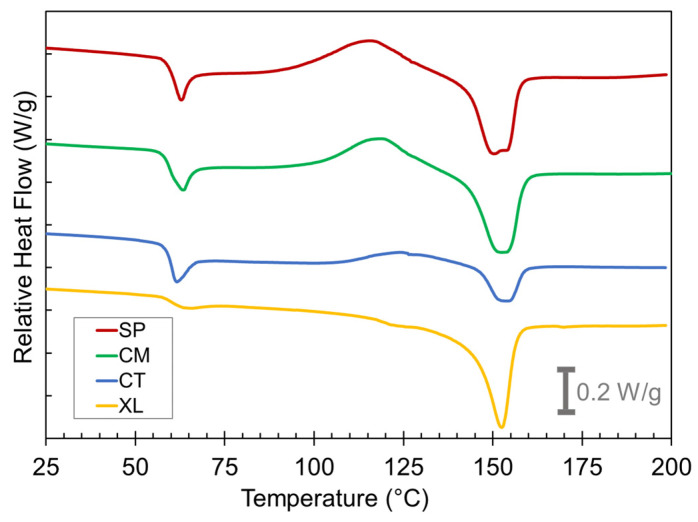
DSC first heat curves for the SP, CM, CT, and XL foams with 6.5 wt% CFA (exo up).

We turned to thermal characterization by DSC to examine the PLA crystal development that occurred in the compression-molded foams. Figure 5 compares the thermograms of the first heat of as-compression-molded foam samples of the 6.5 wt% CFA concentration grouping. The key thermal events occurring during the first heat curves are the glass transition at *T_g_* = 60 °C, cold crystallization at *T_cc_* = 100 °C, and melting at *T_m_* = 150 °C.

The thermogram shape of the XL foams at *T_g_* is a typical step change expected for reversible glass transition whereas the CT, CM, and SP foams record a *T_g_* overshoot peak in their thermograms at ~60 °C. These overshoots were caused by the devitrification of additional mobile amorphous phase PLA in the sample after cooling during the foaming process [25]. The reasoning behind this will be explained later in this section.

Significant cold crystallization exotherms occurred beginning at ~100 °C for the SP and CM foams. Conversely, the CT samples displayed only a shallow cold crystallization peak, and the peak shifted to a higher temperature range than the solid-state-processed samples. The XL samples showed no cold crystallization. These findings indicate that solid-state-processed foams show a higher potential to crystallize whereas the CT and XL samples either have a lower capacity to crystallize or have already crystallized to their full extent before 100 °C. The melt peak characteristics reveal more about which of these is occurring. Clear differences can immediately be seen between the different melting endotherms at *T_m_* ~150 °C. The melting peaks for the solid-state-processed samples were much larger than for the CT sample. This indicates that the solid-state-processed samples underwent a significant level of total crystallization prior to melting, whereas the CT samples were less able to crystallize comprehensively, resulting in a small melting peak. The double peak nature of the SP sample melt peaks has been attributed to reflecting the recrystallization and reorganization process in a previous study [25]. Despite the XL samples also displaying little evidence of cold crystallization, they still had large melting peaks, indicating that any crystallization in the XL foams happened during the initial foaming process rather than the DSC’s first heat run. These contrastive thermogram features between SP, CM, CT, and XL samples were observed at all CFA concentrations.

The differences in the relative latent heat of melting (∆*H_m_*) vs. cold crystallization (∆*H_cc_*) are worth further investigating. Table 1 lists the two latent heats from the first heat thermograms, and further calculates the effective latent heat of “melt crystallization” (∆*H_mc_*) during the respective foaming process, i.e., the measure of the extent that the PLA was able to crystallize during the cooling step of the compression molding after the CFA has been activated [25]. This value was calculated by subtracting ∆*H_cc_* from ∆*H_m_*. Table 1 reveals that in every case, ∆*H_mc_*, ∆*H_cc_*, and ∆*H_m_* are all greater for the SP and CM samples than for the CT samples. The ∆*H_mc_* values for SP and CM foams were recorded in the range of 5.0–8.5 J/g, compared to 0.5–3.0 J/g for CT foams. While there appears to be no significant correlation between CFA concentration and enthalpy values, the higher ranges in SP and CM confirm substantial PLA crystallite development during their cooling process in the compression mold, which led to more effective containment of chemically induced gas in the foams.

Interestingly, the XL foams did not display large cold crystallization peaks but still exhibited significantly large melting curves, suggesting that much of the crystallization occurred during foam cooling, to an extent even larger than those of SP and CM samples. Despite the significant crystallization enhancement caused by crosslinking, Section 3.1 showed how the XL foam void fraction values remain significantly lower than the non-crosslinked analogs in the CM set. This suggests that while a moderate amount of crystallization during foaming is desirable, exemplified by solid-state processing cases, excessive crystallization inhibits foaming by over-stiffening the PLA matrix. A similar inhibition of PLA foaming by excessive crystallinity has also previously been observed in physical foaming contexts [62,64].

### 3.4. Dynamic Mechanical Analysis Results

**Figure 6:**
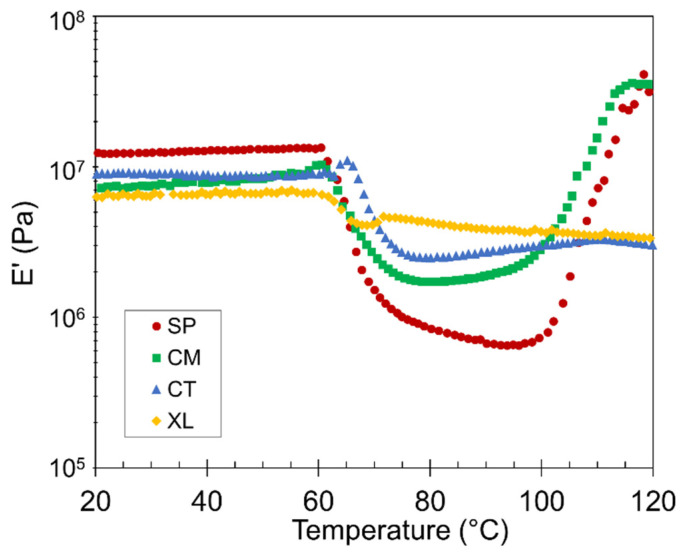
Representative plots of E’ versus temperature for SP, CM, CT, and XL foams with 6.5 wt% CFA.

Temperature ramp DMA was conducted to observe the mechanical properties of the foams as a function of temperature, as well as to verify the crystallization behavior that was inferred from the DSC study above. We first focus on the changes in storage modulus (*E’*) in Figure 6 based on a representative set. The 6.5 wt% CFA samples were selected because their substantial density reductions provided the highest contrast of DMA curves between the four contrastive foam samples within the series. The same thermal transition events and relative *E’* position trends were observed in other series of CFA concentrations. We limit the following discussion to qualitative comparisons.

The stiffness of the PLA foams remained relatively constant from room temperature up to the *T_g_* ~60 °C, above which the foams lose stiffness as their chains become mobile. Note that the relative *E’* positions of the four samples switch between the pre- and post-*T_g_* plateaus in Figure 6. In the region between glass and cold crystallization temperatures, the SP and CM samples exhibited lower *E’* values. With higher void fraction and cell density, the two solid-state-processed foams displayed a suppressed solid-like behavior, especially because their crystallinity during this region was only modest. In contrast, the XL sample did not experience a drastic decrease in stiffness after *T_g_*, as it was supported by the crosslinks and significant crystallinity that had already developed.

The 100–120 °C region corresponds to cold crystallization. A gradual modulus recovery correlates with the increasing number of developing crystals, as the crystalline phase is stiffer than the amorphous component above the *T_g_* [65]. Figure 6 reveals that the solid-state-processed SP and CM samples experienced significant cold crystallization, to a level higher than any CT stiffness value and even surpassing their own original *E’*, having raised their crystalline potential [25]. On the other hand, the CT and XL samples showed little to no cold crystallization, corroborating the DSC results.

**Figure 7:**
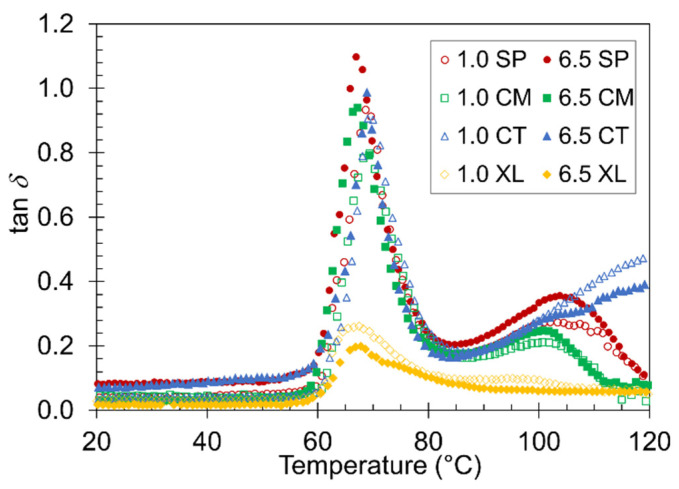
Representative plots of tan *δ* versus temperature for the four contrastive foams with 1.0 wt% and 6.5 wt% CFA.

Often, one of the most valuable properties of foam material is its ability to absorb energy [66,67]. The tan *δ* plot of the temperature ramp DMA can be used to observe the material damping factor of the samples [68,69]. The higher the value of tan *δ* at a given temperature, the more the material will absorb energy [68]. Figure 7 compares tan *δ* curves between the 1.0 wt% and 6.5 wt% CFA series of the four foam sets. A major peak in tan *δ* at *T_g_* associated with PLA devitrification was observed in each sample, as expected from a previous study on compression DMA of polymer foams [70]. The height of the tan *δ* peak varied slightly depending on the pre-foaming processing method. The most noticeable difference was between the XL foams and the SP, CM, and CT foams, which suggests that the XL foams remained too rigid through the *T_g_* and deviated from a typical foam behavior in its mechanical response to the oscillatory motion. A peak height difference was also observed between the 6.5 wt% and 1.0 wt% samples of a given foam set. The fact that the higher CFA content foams constantly displayed a higher damping factor in the SP, CM, and CT sets confirms the effectiveness of employing higher CFA loading in preparing PLA foams. Again, the XL foams did not follow the same trend because their foam structure and rigidity properties are fundamentally different from the other sets.

A second, shallower, and broader tan *δ* peak appeared around 100 °C most distinctly in the solid-state-processed samples. The CT foams showed a continuously gradual increase without peaking, while the XL foams did not show any evidence of a significant second peak. As discussed above with *E’* transitions, the SP, CM, and CT samples developed more liquid-like and damping behaviors above their devitrification points. This typical and desired foam property caused tan *δ* to remain high until cold crystallization occurs in the respective sample, at which point stiff solid-like behavior returns and lowers the damping factor. The CT curve continued to display high tan *δ* due to a lack of cold crystallization. The XL foams were already stiff and crystalline before *T_g_*, causing the tan *δ* curve to remain low.

### 3.5. Static Compression Testing

**Figure 8:**
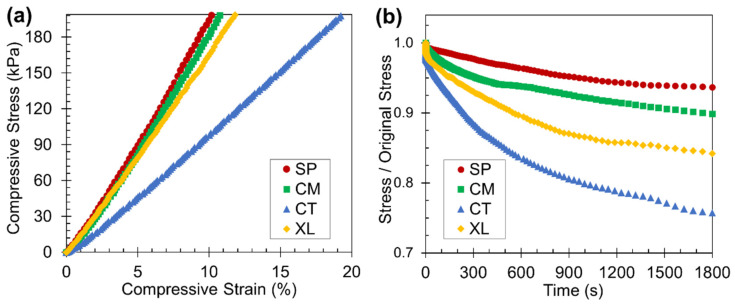
Results from the static compression testing displaying the (**a**) stress-stain curves (truncated at a compressive stress of 200 kPa) and (**b**) stress relaxation curves of the four foam samples with 6.5 wt% CFA at room temperature.

As polymer foams are likely used in practical applications at ambient temperatures, room-temperature static compression tests were carried out to determine the stress-strain relationships and stress relaxation tendencies. Based on the representative foam set of 6.5 wt% CFA, Figure 8a reveals that the SP and CM samples both displayed stress-strain relationships with higher slopes than the CT set. The solid-state-processed PLA foam samples were significantly stiffer and more mechanically robust than manually blended foam samples at room temperature, due to their higher as-molded crystallinity, as observed earlier by the ∆*H_mc_* values. The stiffness difference may also reflect that in the foam morphology, as Section 3.2 established that the solid-state-processed samples displayed higher cell density and more spatially consistent closed cell structure. The XL samples exhibited high stiffness because of their enhanced crystallinity through crosslinking and cryomilling; crosslinking has previously been shown to make PLA stiffer [53]. However, the XL sample’s stress-strain curve had a lower slope than the SSSP and cryomill stress-strain plots perhaps due to the open-cell nature of the crosslinked foam [71].

The stress relaxation results in Figure 8b reveal that SP and CM foams relaxed to a lesser extent than CT foams when subject to a constant initial static load of 200 kPa. In the context of static loading, foams that undergo less stress relaxation are better able to retain their initial shape after being compressed to some reasonable deformation level, enabling sustainable usage in various applications. For certain packaging applications, an ideal foam can be defined as one that continuously applies a consistent force on an object [72], which the solid-state-processed foams exhibit. While the XL foams had high crystalline properties, their open cell foam structure nonetheless caused significant stress relaxation to occur.

## 4. Conclusions

An effective PLA chemical foaming method has been established through solid-state processing, via either SSSP or cryomilling, followed by compression molding. Solid-state-processing PLA achieved foams with void fraction values approximately double those of the control foams (70% versus 35%) and consistently higher cell density. Though unusual, a relatively high CFA loading of around 6 wt% is recommended with solid-state processing, as increasing CFA concentration resulted in a corresponding increase in void fraction up until a plateau value. DSC and DMA findings indicated that the shearing and pulverization effects of solid-state processing resulted in enhanced melt crystallization and cold crystallization enthalpies. Additionally, solid-state-processed foams proved more robust and displayed less stress relaxation than crosslinked and control foam sets, enabling better reusability for sustainable applications. The crosslinked foams, which were also solid-state processed, achieved the highest level of melt crystallization but achieved low void fraction values (~30%) and inconsistent cell density, disproving that combining solid-state processing and crosslinking is an effective strategy for PLA foam development.

In the future, a better understanding of the optimal compression molding foaming heating and cooling rates should be established to ensure the most effective foaming method for PLA foams. One potential route is an in-depth investigation into the interplay between foaming and crystallization at different solidification rates. The chemical foaming method developed in this study complements existing physical foaming methods for PLA and contributes toward the widespread application of sustainable foams in our society.

## Figures and Tables

**Table 1:** Crystallization characteristics extrapolated from DSC first heat curves, with the top number in each cell being ∆*H_m_*, the middle, subtracted number being ∆*H_cc_*, and the resulting number (shaded) being ∆*H_mc_*. Note: all values are reported in J/g.

Processing Type	Enthalpy Type (J/g)	Concentration of CFA (Nominal wt%)					
		0.5 wt%	1.0 wt%	2.0 wt%	3.5 wt%	5.0 wt%	6.5 wt%
SSSP (SP)	∆*H_m_*	15.5	16.1	17.2	15.7	17.1	14.0
	∆*H_cc_*	10.0	9.0	9.6	8.3	9.2	8.0
	∆ *H_mc_*	5.5	7.1	7.6	7.4	7.9	6.0
Cryomill (CM)	∆*H_m_*	14.8	16.6	16.0	14.2	17.2	15.0
	∆*H_cc_*	6.7	8.8	7.5	7.8	12.0	8.6
	∆ *H_mc_*	8.1	7.8	8.5	6.4	5.2	6.4
Control (CT)	∆*H_m_*	3.1	5.3	5.2	5.5	4.4	4.7
	∆*H_cc_*	1.4	2.7	4.7	3.2	3.5	2.2
	∆ *H_mc_*	1.7	2.6	0.5	2.3	0.9	2.5
Crosslinked (XL)	∆*H_m_*	17.4	19.0	16.5	20.5	16.4	17.7
	∆*H_cc_*	2.1	4.7	1.3	2.5	2.0	0.7
	∆ *H_mc_*	15.3	14.3	15.2	18.0	14.4	17.0

## Data Availability

The data presented in this study are available upon request from the corresponding author.

## References

[1] Doyle L., Weidlich I. (2019). Mechanical Behaviour of Polylactic Acid Foam as Insulation under Increasing Temperature. Environ. Clim. Technol..

[2] Nofar M., Park C.B. (2014). Poly (Lactic Acid) Foaming. Prog. Polym. Sci..

[3] Lee C.H., Lee K.-J., Jeong H.G., Kim S.W. (2000). Growth of Gas Bubbles in the Foam Extrusion Process. Adv. Polym. Technol..

[4] Altan M., Cankaya N. (2018). Thermoplastic Foams: Processing, Manufacturing, and Characterization. Recent Research in Polymerization.

[5] Chamas A., Moon H., Zheng J., Qiu Y., Tabassum T., Jang J.H., Abu-Omar M., Scott S.L., Suh S. (2020). Degradation Rates of Plastics in the Environment. ACS Sustain. Chem. Eng..

[6] Song J.H., Murphy R.J., Narayan R., Davies G.B.H. (2009). Biodegradable and Compostable Alternatives to Conventional Plastics. Phil. Trans. R. Soc. B.

[7] Zhu Y., Romain C., Williams C.K. (2016). Sustainable Polymers from Renewable Resources. Nature.

[8] Zhao S., Malfait W.J., Guerrero-Alburquerque N., Koebel M.M., Nyström G. (2018). Biopolymer Aerogels and Foams: Chemistry, Properties, and Applications. Angew. Chem. Int. Ed..

[9] Gama N.V., Soares B., Freire C.S.R., Silva R., Neto C.P., Barros-Timmons A., Ferreira A. (2015). Bio-Based Polyurethane Foams toward Applications beyond Thermal Insulation. Mater. Des..

[10] Dorgan J.R., Lehermeier H., Mang M. (2000). Thermal and Rheological Properties of Commercial-Grade Poly(Lactic Acid)s. J. Polym. Environ..

[11] Cheng Y., Deng S., Chen P., Ruan R. (2009). Polylactic Acid (PLA) Synthesis and Modifications: A Review. Front. Chem. China.

[12] Carothers W.H., Dorough G.L., van Natta F.J. (1932). Studies of Polymerization and Ring Formation. X. The Reversible Polymerization of Six-Membered Cyclic Esters. J. Am. Chem. Soc..

[13] (2009). Test Method for Determining Aerobic Biodegradation of Plastic Materials in the Marine Environment by a Defined Microbial Consortium or Natural Sea Water Inoculum.

[14] Greene J. (2012). PLA and PHA Biodegradation in the Marine Environment.

[15] Larsen Å., Neldin C. (2013). Physical Extruder Foaming of Poly(Lactic Acid)-Processing and Foam Properties. Polym. Eng. Sci..

[16] Rokkonen T., Peltola H., Sandquist D. (2019). Foamability and Viscosity Behavior of Extrusion Foamed PLA–Pulp Fiber Biocomposites. J. Appl. Polym. Sci..

[17] Lee S.-T., Ramesh N.S. (2004). Polymeric Foams: Mechanisms and Materials.

[18] Kmetty Á., Litauszki K., Réti D. (2018). Characterization of Different Chemical Blowing Agents and Their Applicability to Produce Poly(Lactic Acid) Foams by Extrusion. Appl. Sci..

[19] Yuan H., Liu Z., Ren J. (2009). Preparation, Characterization, and Foaming Behavior of Poly(Lactic Acid)/Poly(Butylene Adipate- *Co* -Butylene Terephthalate) Blend. Polym. Eng. Sci..

[20] Seo J.-H., Han J., Lee K.S., Cha S.W. (2012). Combined Effects of Chemical and Microcellular Foaming on Foaming Characteristics of PLA (Poly Lactic Acid) in Injection Molding Process. Polym.-Plast. Technol. Eng..

[21] Matuana L.M., Faruk O., Diaz C.A. (2009). Cell Morphology of Extrusion Foamed Poly(Lactic Acid) Using Endothermic Chemical Foaming Agent. Bioresour. Technol..

[22] Wang J., Zhu W., Zhang H., Park C.B. (2012). Continuous Processing of Low-Density, Microcellular Poly(Lactic Acid) Foams with Controlled Cell Morphology and Crystallinity. Chem. Eng. Sci..

[23] Zhao M., Ding X., Mi J., Zhou H., Wang X. (2017). Role of High-Density Polyethylene in the Crystallization Behaviors, Rheological Property, and Supercritical CO_2_ Foaming of Poly (Lactic Acid). Polym. Degrad. Stab..

[24] Campuzano J.F., Lopez I.D. (2020). Study of the Effect of Dicumyl Peroxide on Morphological and Physical Properties of Foam Injection Molded Poly(Lactic Acid)/Poly(Butylene Succinate) Blends. Express Polym. Lett..

[25] Blumer E.M., Lynch B.B., Fielding A.S., Wakabayashi K. (2019). Crystallinity and Property Enhancements in Neat Polylactic Acid by Chilled Extrusion: Solid-state Shear Pulverization and Solid-state/ Melt Extrusion. Polym. Eng. Sci..

[26] Lynch B.B. (2014). The Crystallization Kinetics of Polylactic Acid (PLA) Processed Through Solid-State/Melt Extrusion. B.S. Thesis.

[27] Brunner P. (2013). Overcoming Sustainability and Energy Challenges in Polymer Science Via Solid-State Shear Pulverization. Ph.D. Thesis.

[28] Ganglani M., Torkelson J.M., Carr S.H., Khait K. (2001). Trace Levels of Mechanochemical Effects in Pulverized Polyolefins. J. Appl. Polym. Sci..

[29] Diop M.F., Torkelson J.M. (2015). Novel Synthesis of Branched Polypropylene via Solid-State Shear Pulverization. Polymer.

[30] Lebovitz A.H., Khait K., Torkelson J.M. (2002). In Situ Block Copolymer Formation during Solid-State Shear Pulverization: An Explanation for Blend Compatibilization via Interpolymer Radical Reactions. Macromolecules.

[31] Tao Y., Lebovitz A.H., Torkelson J.M. (2005). Compatibilizing Effects of Block Copolymer Mixed with Immiscible Polymer Blends by Solid-State Shear Pulverization: Stabilizing the Dispersed Phase to Static Coarsening. Polymer.

[32] Diop M.F., Burghardt W.R., Torkelson J.M. (2014). Well-Mixed Blends of HDPE and Ultrahigh Molecular Weight Polyethylene with Major Improvements in Impact Strength Achieved via Solid-State Shear Pulverization. Polymer.

[33] Tao Y., Kim J., Torkelson J.M. (2006). Achievement of Quasi-Nanostructured Polymer Blends by Solid-State Shear Pulverization and Compatibilization by Gradient Copolymer Addition. Polymer.

[34] Walker A.M., Tao Y., Torkelson J.M. (2007). Polyethylene/Starch Blends with Enhanced Oxygen Barrier and Mechanical Properties: Effect of Granule Morphology Damage by Solid-State Shear Pulverization. Polymer.

[35] Iyer K.A., Lechanski J., Torkelson J.M. (2016). Green Polypropylene/Waste Paper Composites with Superior Modulus and Crystallization Behavior: Optimizing Specific Energy in Solid-State Shear Pulverization for Filler Size Reduction and Dispersion. Compos.-A Appl. Sci. Manuf..

[36] Wakabayashi K., Pierre C., Dikin D.A., Ruoff R.S., Ramanathan T., Brinson L.C., Torkelson J.M. (2008). Polymer-Graphite Nanocomposites: Effective Dispersion and Major Property Enhancement via Solid-State Shear Pulverization. Macromolecules.

[37] Pujari S., Ramanathan T., Kasimatis K., Masuda J., Andrews R., Torkelson J.M., Brinson L.C., Burghardt W.R. (2009). Preparation and Characterization of Multiwalled Carbon Nanotube Dispersions in Polypropylene: Melt Mixing versus Solid-State Shear Pulverization. J. Polym. Sci. B Polym. Phys..

[38] Wakabayashi K., Brunner P.J., Masuda J., Hewlett S.A., Torkelson J.M. (2010). Polypropylene-Graphite Nanocomposites Made by Solid-State Shear Pulverization: Effects of Significantly Exfoliated, Unmodified Graphite Content on Physical, Mechanical and Electrical Properties. Polymer.

[39] Iyer K.A., Torkelson J.M. (2013). Novel, Synergistic Composites of Polypropylene and Rice Husk Ash: Sustainable Resource Hybrids Prepared by Solid-State Shear Pulverization. Polym. Compos..

[40] Miu E.V., Fox A.J., Jubb S.H., Wakabayashi K. (2016). Morphology and Toughness Enhancements in Recycled High-Density Polyethylene (rHDPE) via Solid-State Shear Pulverization (SSSP) and Solid-State/Melt Extrusion (SSME). J. Appl. Polym. Sci..

[41] Khait K., Torkelson J.M. (1999). Solid-State Shear Pulverization of Plastics: A Green Recycling Process. Polym. -Plast. Technol. Eng..

[42] Iyer K.A., Torkelson J.M. (2014). Green Composites of Polypropylene and Eggshell: Effective Biofiller Size Reduction and Dispersion by Single-Step Processing with Solid-State Shear Pulverization. Compos. Sci. Technol..

[43] Iyer K.A., Flores A.M., Torkelson J.M. (2015). Comparison of Polyolefin Biocomposites Prepared with Waste Cardboard, Microcrystalline Cellulose, and Cellulose Nanocrystals via Solid-State Shear Pulverization. Polymer.

[44] Wakabayashi K., Vancoillie S.H.E., Assfaw M.G., Choi D.H., Desplentere F., Van Vuure A.W. (2019). Low-temperature Compounding of Flax Fibers with Polyamide 6 via Solid-state Shear Pulverization: Towards Viable Natural Fiber Composites with Engineering Thermoplastics. Polym. Compos..

[45] Brunner P.J., Torkelson J.M. (2011). Microcrystalline Cellulose Composites of Poly(Lactic Acid)/Poly(Ethylene Glycol) or Polypropylene Created via Solid-State Shear Pulverization. ANTEC Proc..

[46] Henry M.F. (2010). Solid-State Compatibilization of Immiscible Polymer Blends: Cryogenic Milling and Solid-State Shear Pulverization. M.S. Thesis.

[47] Hubert P.J., Kathiresan K., Wakabayashi K. (2011). Filler Exfoliation and Dispersion in Polypropylene/as-Received Graphite Nanocomposites via Cryogenic Milling. Polym. Eng. Sci..

[48] Gabriel M.C., Mendes L.B., de Melo Carvalho B., Pinheiro L.A., Capochi J.D.T., Kubaski E.T., Cintho O.M. (2010). High-Energy Mechanical Milling of Ultra-High Molecular Weight Polyethylene (UHMWPE). Mater. Sci. Forum.

[49] Segura González E.A., Olmos D., González-Gaitano G., Orgaz B., González-Benito J. (2015). Effect of Kaolin Nanofiller and Processing Conditions on the Structure, Morphology, and Biofilm Development of Polylactic Acid. J. Appl. Polym. Sci..

[50] Candau N., Oguz O., León Albiter N., Förster G., Maspoch M.L. (2021). Poly (Lactic Acid)/Ground Tire Rubber Blends Using Peroxide Vulcanization. Polymers.

[51] (2015). Ingeo Biopolymer 2003D Technical Data Sheet.

[52] Bhatti A.S., Goddard R.J., O’Donnell G. (1984). The Thermal Decomposition of Azodicarbonamide. Thermochim. Acta.

[53] Yang S., Wu Z.-H., Yang W., Yang M.-B. (2008). Thermal and Mechanical Properties of Chemical Crosslinked Polylactide (PLA). Polym. Test..

[54] Onffroy P.R., Miu E.V., Confer W.J., Darkes-Burkey C.M., Holler W.C., Wakabayashi K. (2020). Residence Time Distribution and Specific Mechanical Energy in Solid-state Shear Pulverization: Processing-structure-property Relationships in a Chilled Extruder. Polym. Eng. Sci..

[55] Katiyar N.K., Biswas K., Tiwary C.S. (2021). Cryomilling as Environmentally Friendly Synthesis Route to Prepare Nanomaterials. Int. Mater. Rev..

[56] Matuana L.M., Diaz C.A. (2010). Study of Cell Nucleation in Microcellular Poly(Lactic Acid) Foamed with Supercritical CO_2_ through a Continuous-Extrusion Process. Ind. Eng. Chem. Res..

[57] Reglero Ruiz J.A., Vincent M., Agassant J.-F., Sadik T., Pillon C., Carrot C. (2015). Polymer Foaming with Chemical Blowing Agents: Experiment and Modeling. Polym. Eng. Sci..

[58] Miltz J., Ramon O. (1986). Characterization of Stress Relaxation Curves of Plastic Foams. Polym. Eng. Sci..

[59] Khemani K.C. (1997). Polymeric Foams: Science and Technology.

[60] Jang L.K., Fletcher G.K., Monroe M.B.B., Maitland D.J. (2020). Biodegradable Shape Memory Polymer Foams with Appropriate Thermal Properties for Hemostatic Applications. J. Biomed. Mater. Res..

[61] Wang L., Hikima Y., Ohshima M., Yusa A., Yamamoto S., Goto H. (2017). Development of a Simplified Foam Injection Molding Technique and Its Application to the Production of High Void Fraction Polypropylene Foams. Ind. Eng. Chem. Res..

[62] Zhai W., Ko Y., Zhu W., Wong A., Park C.B. (2009). A Study of the Crystallization, Melting, and Foaming Behaviors of Polylactic Acid in Compressed CO_2_. Int. J. Mol. Sci..

[63] Barzegari M.R., Rodrigue D. (2009). The Effect of Injection Molding Conditions on the Morphology of Polymer Structural Foams. Polym. Eng. Sci..

[64] Standau T., Zhao C., Murillo Castellón S., Bonten C., Altstädt V. (2019). Chemical Modification and Foam Processing of Polylactide (PLA). Polymers.

[65] Liu G., Zhang X., Wang D. (2014). Tailoring Crystallization: Towards High-Performance Poly(Lactic Acid). Adv. Mater..

[66] Tomin M., Kmetty Á. (2022). Polymer Foams as Advanced Energy Absorbing Materials for Sports Applications—A Review. J. Appl. Polym. Sci..

[67] Yang B., Zuo Y., Chang Z. (2021). Evaluation of Energy Absorption Capabilities of Polyethylene Foam under Impact Deformation. Materials.

[68] Shaid Sujon M.A., Islam A., Nadimpalli V.K. (2021). Damping and Sound Absorption Properties of Polymer Matrix Composites: A Review. Polym. Test..

[69] Bizhani H., Katbab A.A., Lopez-Hernandez E., Miranda J.M., Lopez-Manchado M.A., Verdejo R. (2020). Preparation and Characterization of Highly Elastic Foams with Enhanced Electromagnetic Wave Absorption Based On Ethylene-Propylene-Diene-Monomer Rubber Filled with Barium Titanate/Multiwall Carbon Nanotube Hybrid. Polymers.

[70] Zhang L., Jeon H.K., Malsam J., Herrington R., Macosko C.W. (2007). Substituting Soybean Oil-Based Polyol into Polyurethane Flexible Foams. Polymer.

[71] Song P., Zhang Y., Luo Y., Liao X., Tang W., Yang J., Tian C., Li G. (2022). Design of Lightweight Silicone Rubber Foam for Outstanding Deformation Recoverability Based on Supercritical CO_2_ Foaming Technology. J. Mater. Sci..

[72] Miltz J., Ramon O. (1990). Energy Absorption Characteristics of Polymeric Foams Used as Cushioning Materials. Polym. Eng. Sci..

